# Evaluation of the safety and efficiency of novel metallic implant scaler tips manufactured by the powder injection molding technique

**DOI:** 10.1186/s12903-017-0396-z

**Published:** 2017-07-11

**Authors:** Kyung A. Chun, Kee-Yeon Kum, Woo-Cheol Lee, Seung-Ho Baek, Hae-Won Choi, Won-Jun Shon

**Affiliations:** 1 Department of Conservative Dentistry, Dental Research Institute and School of Dentistry, Seoul National University, 101 Daehak-ro, Jongno-gu, Seoul, 03080 South Korea; 2 Department of Conservative Dentistry, Korea University Anam Hospital, 73, Inchon-ro, Seongbuk-gu, Seoul, 02841 South Korea; 3 Department of Orthodontics, The Institute of Oral Health Science, Samsung Medical Center, Sungkyunkwan University School of Medicine, Seoul, South Korea; 4 Department of Dental Biomaterials Science, Dental Research Institute and School of Dentistry, Seoul National University, 101 Daehak-ro, Jongno-gu, Seoul, 03080 South Korea

**Keywords:** Implant scaler tip, Novel metal, Powder injection molding (PIM), Titanium surface, Efficiency

## Abstract

**Background:**

Although many studies have compared the properties of ultrasonic scaling instruments, it remains controversial as to which is most suitable for implant scaling. This study evaluated the safety and efficiency of novel metallic ultrasonic scaler tips made by the powder injection molding (PIM) technique on titanium surfaces.

**Methods:**

Mechanical instrumentation was carried out using four types of metal scaler tips consisting of copper (CU), bronze (BR), 316 L stainless steel (316 L), and conventional stainless steel (SS) tips. The instrumented surface alteration image of samples was viewed with scanning electron microscope (SEM) and surface profile of the each sample was investigated with confocal laser scanning microscopy (CLSM). Arithmetic mean roughness (Ra) and maximum height roughness (Rmax) of titanium samples were measured and dissipated power of the scaler tip was estimated for scaling efficiency.

**Results:**

The average Ra values caused by the 316 L and SS tip were about two times higher than those of the CU and BR tips (*p* < 0.05). The Rmax value showed similar results. The efficiency of the SS tip was about 3 times higher than that of CU tip, the 316 L tip is about 2.7 times higher than that of CU tip, and the BR tip is about 1.2 times higher than that of CU tip.

**Conclusions:**

Novel metallic bronze alloy ultrasonic scaler tip minimally damages titanium surfaces, similar to copper alloy tip. Therefore, this bronze alloy scaler tip may be promising instrument for implant maintenance therapy.

## Background

Peri-implantitis caused by plaque accumulation is a major risk factor for failure of dental implant therapy [1]. Although patients can remove plaques with standard prophylactic agents, professional cleaning of the implant using various instruments is required during the implant maintenance phase. However, routine prophylactic procedures using a conventional stainless steel instrument can cause damage to implant surfaces over time and increase the potential for plaque accumulation [2]. The progression of peri-implantitis was more pronounced in implants with a moderately rough surface than in those with a polished surface [3, 4]. It has been suggested that nonmetallic instruments such as rubber cups, plastic curettes, titanium curettes, and air-powder abrasive systems are suitable tools for implant maintenance [5–10]. These non-metallic instruments have been shown to be effective for supra gingival removal of calculus and plaque on implant surfaces without the risk of damage [11–13]. However, the application of air-powder abrasive systems has been reported to be associated with an increased risk of emphysema [14]. It has also been reported that nonmetallic carbon fiber tips (Vector ultrasonic scaler) are not suitable for decontaminating titanium surfaces [15]. Moreover, plastic-covered ultrasonic scalers have been shown to leave behind plastic deposits on the implant surface [16]. Although many studies have compared the properties of ultrasonic scaling instruments, it remains controversial as to which is most suitable for implant scaling. While capable of efficient removal of plaque and calculus, the conventional metal tips of sonic and ultrasonic scalers seem to induce considerable change to titanium surfaces [17]. However, this study did not consider the mechanical properties of scaler tips, such as fracture resistance or wear resistance, nor compare their efficiency.

Injection molding is one of the most interesting processing techniques for shaping metals and ceramics near net shapes with reasonably tight tolerance and good surface finish [18]. The process is generally viable for all shapes, which can be formed using plastic injection molding. This technique allows for the mass production of metal and ceramic parts with complicated shapes while ensuring dimensional reproducibility and near-net-shape formation [19]. Very fine metal powder combined with binder material is injected into a die. The part is ejected, the binder is melted or dissolved, and the part is vacuum sintered. This technique produces parts with a theoretical density of 92% [20].

Recently, a novel ultrasonic scaler tip made mainly of copper alloy showed superior results for titanium surface scaling [21, 22]. In addition to copper alloy, bronze and 316 L, which are lower in hardness than titanium, could be considered candidates for implant scaling instruments. Until now, it has been difficult to create scaling instruments with these candidate metals using the machining process. This study introduces the powder injection molding (PIM) technique, which enables relatively ductile metals to be adequately shaped for a scaler tip. The purpose of this study was to evaluate the safety and efficiency of novel metallic ultrasonic scaler tips made by the PIM technique on titanium surfaces.

## Methods

### Ultrasonic scaler tip

**Fig. 1:**
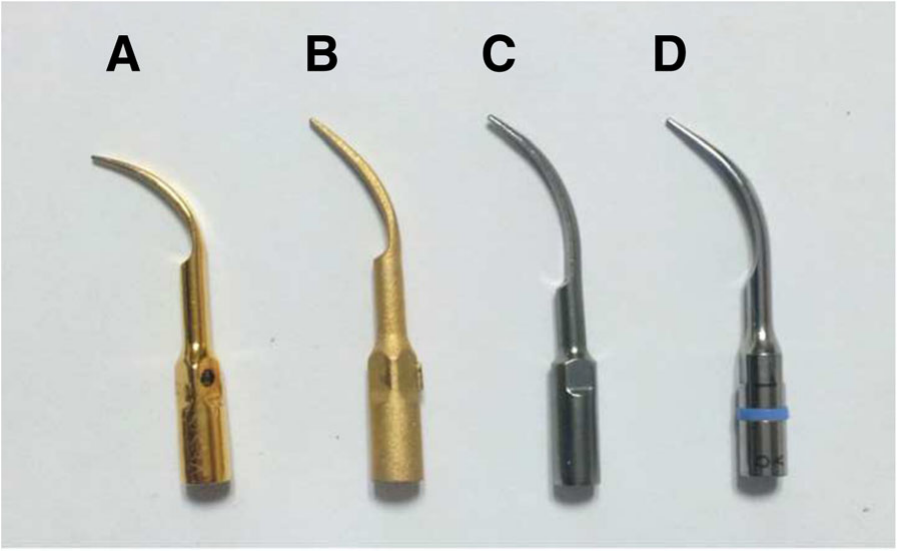
Four types of metal scaler tips used in this study. **a** Copper metallic implant tip (B&L Biotec, Ansan, Korea) (**b**) Bronze metallic implant tip (Cetatec, Sachun, Korea) (**c**) 316 L stainless steel implant tip (Cetatec, Sachun, Korea) (**d**) Conventional stainless steel (Satelec, Merignac, France)

Four types of metal scaler tips consisting of copper (B&L Biotec, Ansan, Korea) (CU) (Fig. 1a), bronze (Cetatec, Sachun, Korea) (BR) (Fig. 1b), 316 L stainless steel (Cetatec, Sachun, Korea) (316 L) (Fig. 1c), and conventional stainless steel (Satelec, Merignac, France) (SS) (Fig. 1d) tips were used. The bronze and the 316 L stainless steel tips were experimental tips manufactured using the PIM technique and had a shape similar to other commercial tips. The manufacturer information and specifications for tips are shown in Table 1. The Vickers hardness value of each tip was measured from the polished surface using a Micro-Hardness tester (HMV-2, Shimadzu, Japan).

**Table 1:** Physical properties of the materials used in this study

	CU	BR	316 L	SS	Ti (VI)
Hardness (HV)	90	120	190	537	280
Density (g/cm^3^)	8.7	8.6	7.6	8.0	4.51^a^
Modulus of Elasticity (GPa)	103	115	193	224	105^a^

The physical properties of scaler tips were provided by manufacturer

^a^Material Property Data, Titanium Grade 4 (http://matweb.com/search/DataSheet.aspx?MatGUID=4b86c47a545247afae3da55d62381f89)

### Fabrication of the samples

Forty (10 per group) pure titanium discs (Grade IV) with a diameter of 10 mm and a thickness of 10 mm were bonded on an epoxy resin block and polished with #4000 grit SiC abrasive paper (Struers A/S, Ballerup, Denmark). The Vickers hardness value of the titanium alloy was also measured after the polishing procedure.

### Scratch test

**Fig. 2:**
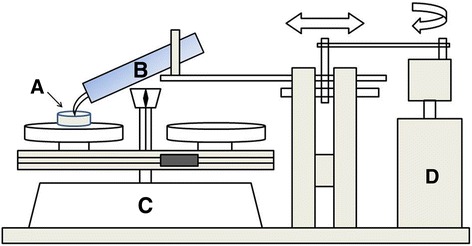
Schematic diagram of the ultrasonic scaling apparatus. **a** = specimen; **b** = ultrasonic scaler; **c** = double-pan balance; **d** = motor with control box

An experimental apparatus similar to the apparatus described by Dentkos et al. was manufactured [23]. A schematic diagram of the apparatus is shown in Fig. 2. The samples were placed on a double pan balance (Ohaus Medical Trip Medical Balance1550-SD, Ohaus Co., Pine Brook, NJ, USA) using a magnetic mold. Each scaling tip was angled at approximately 30° to the polished surface sample. Standardized 3-mm horizontal movement (2 Hz cycle) of the tip was achieved with a constant force of 40 g produced by the vertical movement of the counter-weighed balance. All scaler tips were used for 30 s on 40% of full power. All instrumentation was performed by one investigator. The untreated adjacent surfaces served as control groups (Con). All samples were rinsed in running tap water and cleaned in an ultrasonic bath for 20 min and then dried with compressed air.

### Surface analysis

#### Scanning electron microscope

The instrumented surface characteristics were viewed with a scanning electron microscope (SEM). All titanium discs coated with silver were introduced into the vacuum chamber of a field emission scanning electron microscope (FE-SEM, S-4700, HITACHI, Tokyo, Japan) at an accelerating voltage of 15 kV and observed at 100× and 500× magnification.

#### Confocal laser scanning microscope

Confocal laser scanning microscopy (CLSM, LSM 5 Pascal, Carl Zeiss Microscopy, Göttingen, Germany) was performed to measure the depths and widths of the scratches in the Cu, Br, 316 L, and SS groups. A 543-nm (1-mW) HeNe laser was used as a light source, and the samples were observed at 100× magnification. The measuring area was 920 μm × 920 μm, and the height of the z-stack was 80 μm in 1.6 μm intervals.

The CLSM images were analyzed using a Zeiss LSM Image Examiner Ver. 3.1 (Carl Zeiss, Göttingen, Germany). After drawing a line that passed through the middle of the confocal image, the surface roughness of the line was observed. The arithmetic mean roughness (Ra) and the maximum roughness depth (Rmax) of the titanium samples were measured using CLSM.

The means and standard deviations of Ra and Rmax were calculated for each group after measurement. For the statistical analysis, the results were evaluated using Kruskal–Wallis with Duncan grouping procedures for pair-wise comparisons. Differences at *P* < 0.05 were considered statistically significant (IBM SPSS Version 20, SPSS Inc., Chicago, IL, USA).

### Efficiency test

#### Efficiency calculation

**Fig. 3:**
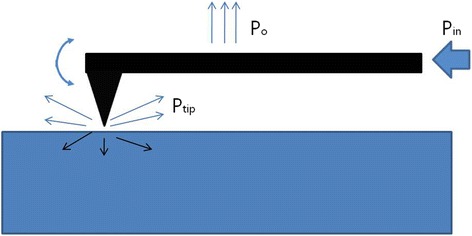
A diagrammatic model of the steady-state motion of an AFM cantilever with the scaler tip. (Po = power dissipated by the body of the cantilever, Ptip = power of dissipation localized to tip, Pin = power of input)

The comparison of efficiency (power) calculations among various scaler tips has been previously proposed [21]. A model of the steady-state motion of an AFM cantilever (Fig. 3) was employed under the assumption that the motion of the scaler tips can be simplified into harmonic oscillation and that the dimensions of the scaler tips are almost the same [24]. We then obtain the following brief formula on the power (efficiency) ratio:

$\frac{\overline{{\mathrm{P}}_{\mathrm{tip}}^{\mathrm{A}}}}{\overline{{\mathrm{P}}_{\mathrm{tip}}^{\mathrm{B}}}}\sim \frac{\sqrt{{\mathrm{E}}_{\mathrm{A}}^3/{\uprho}_{\mathrm{A}}}}{\sqrt{{\mathrm{E}}_{\mathrm{B}}^3/{\uprho}_{\mathrm{B}}}}$ (P: Dissipated power ratio, E: Elastic modulus, ρ: Density).

#### Pre-clinical efficiency evaluation

Cellophane tape with 58 μm thickness (3 M scotch tape) was punched using a dental rubber dam puncher with a diameter of 2 mm and was attached to the surface of a titanium disk. The tape was coated with nail varnish (COLOR AND NATURE®, NATURE REPUBLIC, Seoul, Korea) and the tape removed after the varnish hardened. An area of varnish 2 mm in diameter remained on the surface of the titanium disk and was removed using one of four experimental tips by one operators (*n* = 32 per group). The time taken to remove the varnish was measured to two decimal points. The intra-examiner reliability for the time measurements was assessed using the intraclass correlation coefficient (ICC). The ICC value for the time measurements ranged from 0.91 to 0.95, demonstrating high reliability for all the parameters assessed. Individual mean values and standard deviations were calculated. Comparisons between groups were performed using a one-way analysis of variance (ANOVA) with a Duncan *post-hoc* test. Statistical significance was predetermined as *p* < 0.05. Statistical analyses were performed using SPSS statistical software (IBM SPSS Version 20).

## Results

### Scratch test

#### Changes in surface texture

**Fig. 4:**
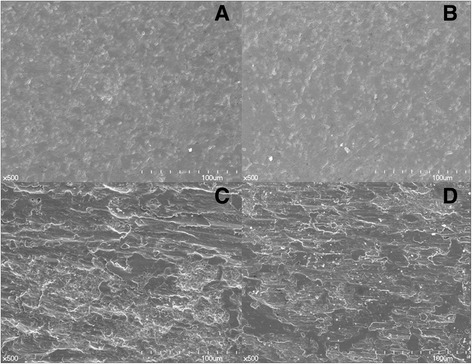
SEM images of specimens after scaling with each experimental tip. **a** Copper metallic implant tip (CU) (**b**) Bronze metallic implant tip (BR) (**c**) 316 L stainless steel implant tip (316 L) (**d**) Conventional stainless steel (SS)

SEM images of each group are shown in Fig. 4. No surface alterations were observed following the use of CU (Fig. 4a) or BR (Fig. 4b) tips, although some smoothening did occur. The use of 316 L (Fig. 4c) and SS (Fig. 4d) tips clearly resulted in scraping of the titanium surfaces and loss of their original texture, leading to increased surface roughness.

### Roughness analysis

**Fig. 5:**
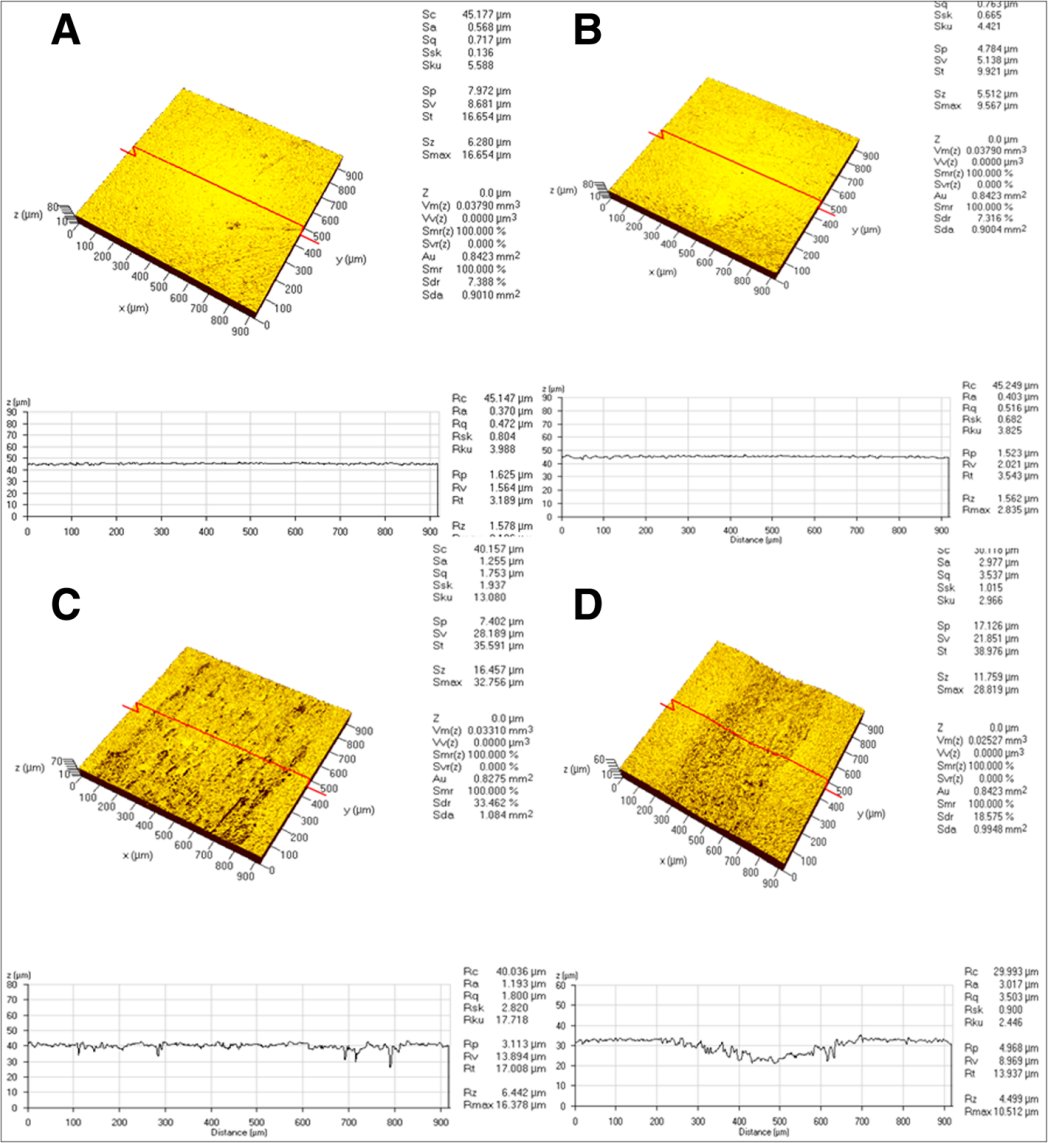
Confocal Laser scanning microscope image of titanium specimens after scaling with each experimental tip. **a** Copper metallic implant tip (CU) (**b**) Bronze metallic implant tip (BR) (**c**) 316 L stainless steel implant tip (316 L) (**d**) Conventional stainless steel (SS)

**Fig. 6:**
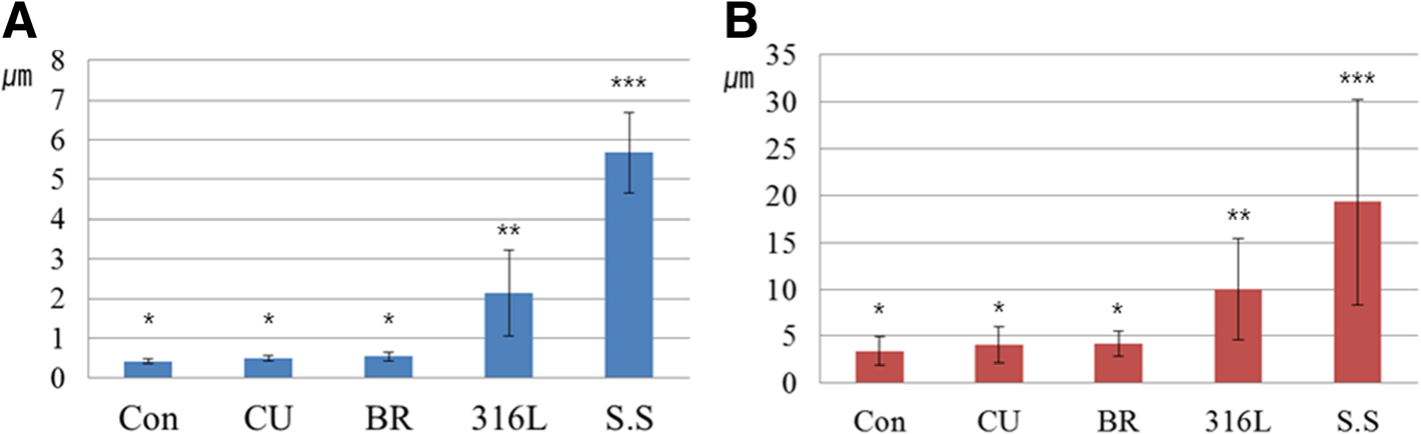
The average roughness (Ra) and maximum height roughness (Rmax) of titanium disk after instrumentation. Copper metallic implant tip (CU), Bronze metallic implant tip (BR), 316 L stainless steel implant tip (316 L), Conventional stainless steel (SS). Same superscript letter means no statistical difference (*p* > 0.05)

Minor surface alterations caused by the CU (Fig. 5a) or BR (Fig. 5b) tips were observed, but considerable changes were observed following the use of the 316 L (Fig. 5c) or SS (Fig. 5d) tips. The average Ra values after instrumentation increased in the order of Con (0.4 μm), CU (0.5 μm), BR (0.5 μm), 316 L (2.1 μm) and SS (5.7 μm) (Fig. 6a). Average Rmax values also increased in a similar order (Fig. 6b). They increased in the order of Con (3.4 μm), CU (4.1 μm), BR (4.2 μm), 316 L (10.0 μm) and SS (19.3 μm). Statistical analysis of the Ra and Rmax values revealed significant differences among the groups (*p* < 0.05). The Con, CU and BR groups showed no statistical differences with each other. The average Ra value of 316 L group was statistically higher than those of the Con, CU and BR ones, and lower than that of SS one (*p* < 0.05).

### Efficiency test

#### Dissipated power calculation

Table 2 showed the scaling efficiency as a dissipated power ratio between the various scaler tips. In this steady-state motion model, the SS group showed the highest dissipated power ratio compared with the other scaler tips. About the calculated dissipated power ratio, SS tip was 3.3, 316 L tip was 2.7, BR tip was 1.2 times higher than CU tip.

**Table 2:** Calculated dissipated power ratio between the various scaler tips

Materials	Power ratio
CU	1
BR	1.2
316 L	2.7
SS	3.3

*CU* Copper, *BR* Bronze, 316 L: 316 L stainless steel, SS: conventional stainless steel

### Pre-clinical efficiency evaluation

The average time taken to remove the nail varnish on the titanium disc is shown in Table 3. The CU and BR groups required significantly more time to remove the nail varnish than the SS group (*P* < 0.05). The 316 L group, which was between the two other groups, showed no significant differences compared to the other groups (*P* < 0.05).

**Table 3:** The average time taken to remove the nail varnish

Group	Time (s)
CU	5.7 (2.1)^a^
BR	5.8 (2.7)^a^
316 L	5.1 (1.3)^a,b^
SS	4.6 (0.7)^b^

*CU* Copper, *BR* Bronze, 316 L: 316 L stainless steel, *SS* conventional stainless steel

The numbers in parenthesis are standard deviations

Same superscript letters mean that there is no statistical difference (*p* > 0.05)

## Discussion

Instruments for cleaning dental implants should be efficient and durable while inflicting minimal damage to the implant surface. Metal instruments have several advantages in that they leave no deposits and have physical properties that are superior to nonmetallic instruments, which tend to be fragile. The copper alloy showed the most reliable results as a replacement for conventional nonmetallic materials in terms of safety and efficiency [21, 22, 25]. The hardness of CU tip (90 HV) and BR tip (120 HV) and 316 L tip (190 HV) are lower than those of titanium disc (280 HV). Bronze is generally harder and less malleable than pure metallic copper. 316 L stainless steels have a range of favorable mechanical properties, including good corrosion resistance, high strength under elevated temperatures, excellent ductility, and good weldability [26]. It has been reported that the hardness of the scaler tip may influence the damage inflicted on titanium surfaces more than the application method [25]. Therefore, we suggested that experimental metal tips made by PIM technique with bronze or 316 L stainless steel would not damage the titanium surface after scaling. From this study, both SEM and CLSM image analyses showed little surface alteration of the titanium after the use of the CU and BR tips, whereas there was considerable change after instrumentation with the 316 L and SS tips. Although the hardness of the 316 L tip is lower than that of titanium disc, the calculated dissipated power ratio of 316 L tip was 2.7 times higher than CU tip (Table 2). So 316 L tip could make surface changes on titanium disc.

A straightforward comparison of the performance between the scaler tips is difficult because the efficiency of the scaler tip depends on various factors such as the material, design, frequency-generating vibration, power, water flow rate, contact angle, and load. Likewise, there is significant variability in the vibration of ultrasonic scalers, even between tips of the same design [27]. For these reasons, in the present study, each specimen was evaluated under standardized conditions as similar to clinical situations as possible. And we employed the model of steady-state motion of a AFM cantilever to explain the power delivered from the driver (the oscillator) to the tip end, based on the assumption that the scaler tips were moving and tapping on the sample surface at equilibrium.

We used the Ra value to measure the safety of the scaler tip for the implant surface in the experiment. Although the average roughness (Ra) parameter is usually used to express the potential initial microbial adhesion to the surfaces of dental implants, it’s not the only factor of microbial adhesion. Also other factors such as the distance from the microbes, the surface chemistry, and the design features of the implant-abutment configuration should be evaluated as well [28–31].

The pre-clinical efficiency test showed that the CU and BR groups took longer to remove the nail varnish than the SS group (*P* < 0.05). Even though there was no significant difference, the 316 L group was able to remove the nail varnish more rapidly than the CU and BR groups. The CU and BR tips appeared more secure but less efficient than the 316 L and SS tips. Since copper and bronze have lower elastic moduli than stainless steel, the elasticity of the metals likely absorbed the vibration of the ultrasonic device. As a result, the oscillation amplitudes of the copper tip and the bronze tip were lower than that of the stainless steel tip, and appeared to be the main cause of the decrease in removal efficiency. In our other study, we have confirmed the results [32]. These are in accord with the outcomes of the power ratio calculation.

In introduction, plastic-covered ultrasonic scaler tip was mentioned to leave plastic deposits on the implant surface. It is also possible that any material softer than titanium may leave remnants of itself on the treated surface. Since the tips used in this experiment also had a lower hardness than titanium, further studies would be needed to determine whether they leave remnants on the titanium surface.

The balance between safety and efficiency is difficult to maintain. However, a previous study demonstrated that the efficiency of a novel metallic copper scaler tip was about 90 times higher than that of a carbon plastic scaler tip [21]. Furthermore, it has been shown that a BR scaler tip made by the PIM technique had safety and efficiency comparable with copper alloy scaler tip and thus has the potential to replace non-metallic instruments since it features superior physical properties such as resistance to fracture and wear.

## Conclusions

Within the limitations of the present study, a novel metallic bronze alloy ultrasonic scaler tip fabricated using the PIM technique minimally damages titanium surfaces and is more durable against fractures and wear compared with copper alloy tips. Therefore, these novel metallic implant scaler tips may be promising for implant maintenance therapy.

## Abbreviations

316 L: 316 L stainless steel
BR: Bronze
CU: Copper
PIM: Powder injection molding
Ra: Arithmetic mean roughness
Rmax: Maximum roughness depth
SS: Stainless steel

## References

[1] Berglundh T, Lindhe J, Marinello C, Ericsson I, Liljenberg B (1992). Soft tissue reaction to de novo plaque formation on implants and teeth. An experimental study in the dog. Clin Oral Implants Res.

[2] Quirynen M, van der Mei HC, Bollen CM, Schotte A, Marechal M, Doornbusch GI (1993). An in vivo study of the influence of the surface roughness of implants on the microbiology of supra- and subgingival plaque. J Dent Res.

[3] Berglundh T, Gotfredsen K, Zitzmann NU, Lang NP, Lindhe J (2007). Spontaneous progression of ligature induced peri-implantitis at implants with different surface roughness: an experimental study in dogs. Clin Oral Implants Res.

[4] Quirynen M, Marechal M, Busscher HJ, Weerkamp AH, Darius PL, van Steenberghe D (1990). The influence of surface free energy and surface roughness on early plaque formation. An in vivo study in man. J Clin Periodontol.

[5] Bailey GM, Gardner JS, Day MH, Kovanda BJ (1998). Implant surface alterations from a nonmetallic ultrasonic tip. J West Soc Periodontol Periodontal Abstr.

[6] Barnes CM, Fleming LS, Mueninghoff LA (1991). SEM evaluation of the in-vitro effects of an air-abrasive system on various implant surfaces. Int J Oral Maxillofac Implants.

[7] Ruhling A, Kocher T, Kreusch J, Plagmann HC (1994). Treatment of subgingival implant surfaces with Teflon-coated sonic and ultrasonic scaler tips and various implant curettes. An in vitro study. Clin Oral Implants Res.

[8] Thomson-Neal D, Evans GH, Meffert RM (1989). Effects of various prophylactic treatments on titanium, sapphire, and hydroxyapatite-coated implants: an SEM study. Int J Periodontics Restor Dent.

[9] Kawashima H, Sato S, Kishida M, Yagi H, Matsumoto K, Ito K (2007). Treatment of titanium dental implants with three piezoelectric ultrasonic scalers: an in vivo study. J Periodontol.

[10] Sato S, Kishida M, Ito K (2004). The comparative effect of ultrasonic scalers on titanium surfaces: an in vitro study. J Periodontol.

[11] Mengel R, Buns CE, Mengel C, Flores-de-Jacoby L (1998). An in vitro study of the treatment of implant surfaces with different instruments. Int J Oral Maxillofac Implants..

[12] Ramaglia L, di Lauro AE, Morgese F, Squillace A (2006). Profilometric and standard error of the mean analysis of rough implant surfaces treated with different instrumentations. Implant Dent.

[13] Fox SC, Moriarty JD, Kusy RP (1990). The effects of scaling a titanium implant surface with metal and plastic instruments: an in vitro study. J Periodontol.

[14] Van de Velde E, Thielens P, Schautteet H, Vanclooster R (1991). Subcutaneous emphysema of the oral floor during cleaning of a bridge fixed on an IMZ implant. Case report. Rev Belge de Med Dent.

[15] Schwarz F, Rothamel D, Sculean A, Georg T, Scherbaum W, Becker J (2003). Effects of an Er:YAG laser and the vector ultrasonic system on the biocompatibility of titanium implants in cultures of human osteoblast-like cells. Clin Oral Implants Res.

[16] Mann M, Parmar D, Walmsley AD, Lea SC (2012). Effect of plastic-covered ultrasonic scalers on titanium implant surfaces. Clin Oral Implants Res.

[17] Hallmon WW, Waldrop TC, Meffert RM, Wade BW (1996). A comparative study of the effects of metallic, nonmetallic, and sonic instrumentation on titanium abutment surfaces. Int J Oral Maxillofac Implants.

[18] Lee SY (2004). Sintering behavior and mechanical properties of injection-molded zirconia powder. Ceram Int.

[19] Lin SIE (2001). Near-net-shape forming of zirconia optical sleeves by ceramics injection molding. Ceram Int.

[20] Moballegh L, Morshedian J, Esfandeh M (2005). Copper injection molding using a thermoplastic binder based on paraffin wax. Mater Lett.

[21] Baek SH, Shon WJ, Bae KS, Kum KY, Lee WC, Park YS (2012). Evaluation of the safety and efficiency of novel metallic ultrasonic scaler tip on titanium surfaces. Clin Oral Implants Res.

[22] Seol HW, Heo SJ, Koak JY, Kim SK, Baek SH, Lee SY (2012). Surface alterations of several dental materials by a novel ultrasonic scaler tip. Int J Oral Maxillofac Implants..

[23] Dentkos TR, Berzins DW (2008). Evaluation of cutting efficiency of orthograde ultrasonic tips by using a nonstatic model. J Endod.

[24] Cleveland JAB, Schmid A, Elings V (1998). Energy dissipation in tapping mode atomic force microscopy. Appl Phys Lett.

[25] Unursaikhan O, Lee JS, Cha JK, Park JC, Jung UW, Kim CS, Cho KS, Choi SH (2012). Comparative evaluation of roughness of titanium surfaces treated by different hygiene instruments. J Periodontal Implant Sci.

[26] Otero E, Pardo A, Utrilla MV, Saenz E, Alvarez JF (1998). Corrosion behaviour of aisi 304L and 316L stainless steels prepared by powder metallurgy in the presence of sulphuric and phosphoric acid. Corros Sci.

[27] Lea SC, Walmsley AD (2009). Mechano-physical and biophysical properties of power-driven scalers: driving the future of powered instrument design and evaluation. Periodontol.

[28] Burgers R, Gerlach T, Hahnel S, Schwarz F, Handel G, Gosau M (2010). In vivo and in vitro biofilm formation on two different titanium implant surfaces. Clin Oral Implants Res.

[29] Elter C, Heuer W, Demling A, Hannig M, Heidenblut T, Bach FW, Stiesch-Scholz M (2008). Supra- and subgingival biofilm formation on implant abutments with different surface characteristics. Int J Oral Maxillofac Implants..

[30] O'Mahony A, MacNeill SR, Cobb CM (2000). Design features that may influence bacterial plaque retention: a retrospective analysis of failed implants. Quintessence Int.

[31] Subramani K, Jung RE, Molenberg A, Hammerle CH (2009). Biofilm on dental implants: a review of the literature. Int J Oral Maxillofac Implants.

[32] Chun KA, Lee IB, Lim BS, Lee JC, Baek SH (2013). Oscillation amplitude of four different metal scaler tips for implants under various load conditions. J Korean Soc Dent Mater.

